# Tissue-engineered Maxillofacial Skeletal Defect Reconstruction by 3D Printed Beta-tricalcium phosphate Scaffold Tethered with Growth Factors and Fibrin Glue Implanted Autologous Bone Marrow-Derived Mesenchymal Stem Cells

**DOI:** 10.25122/jml-2020-0044

**Published:** 2020

**Authors:** Manju Ananthakrishnan Nair, Khadar Vali Shaik, Adiseshu Kokkiligadda, Harsha Gorrela

**Affiliations:** 1. Department of Oral and Maxillofacial Surgery, PAHER University, Udaipur, India; 2. Department of Biotech Engineering, Virchow Biotech Private Limited, Hyderabad, India; 3. Department of Oral and Maxillofacial Surgery, MNR Dental College and Hospital, Hyderabad, India

**Keywords:** Beta tricalcium phosphate (β-TCP) scaffolds, Human Mesenchymal Stem Cells (HMSCs), Fibrin glue (FG), Hounsfield units (HU), tissue engineering, reconstruction, Hydroxyapatite (HA)

## Abstract

The study aimed to investigate whether a 3D printed beta-tricalcium phosphate (β-TCP) scaffold tethered with growth factors and fibrin glue implanted autologous bone marrow-derived mesenchymal stem cells would provide a 3D platform for bone regeneration resulting in new bone formation with plasticity. Twenty 3D printed β-TCP scaffolds, ten scaffolds engrained with osteogenic mesenchymal stem cells with fibrin glue (group A), and ten scaffolds used as a control group with β-TCP scaffold and fibrin glue inoculation only (group B) were included in the study. Cell infiltration, migration, and proliferation of human osteogenic stem cells on the scaffolds were executed under both static and dynamic culture conditions. Each scaffold was examined post culture after repeated changes in the nutrient medium at 2, 4 or 8 weeks and assessed for opacity and formation of any bone-like tissues macroscopic, radiographic, and microscopic evaluation. Significant changes in all the prerequisite parameters compiled with an evaluated difference of significance showing maxillofacial skeletal repair via tissue engineering by β-TCP scaffold and MSCs remains will be the most promising alternative to autologous bone grafts and numerous modalities involving a variety of stem cells, growth factors from platelet-rich fibrin.

## Introduction

Traumatic injuries to the maxillofacial skeleton, chronic infections of dental origin, and surgical removal of benign or malignant tumors cause defects in the facial anatomy. Reconstruction of these defects has been an endeavor to the majority of maxillofacial surgeons to improve aesthetics and function. The gold standard care of maxillofacial skeletal reconstruction was esteemed to be provided by autogenous bone transplantation, mostly free vascularized tissue transfer in patients with a history of major ablative surgeries, as the three essential elements of bone regeneration (osteoconduction, osteoinduction and osteogenicity) are attainable. The knowledge of bioengineering, biology, cell transplantation, and material science in tissue engineering methods can accustom as an alternative that might offer a next step in the evolution of reconstruction of bone as it applies to construct biological substitutes that can restore and maintain normal function in an injured and diseased bone. Integrated beta-tricalcium phosphate (β-TCP) designed in 3D scaffolds and exogenous mesenchymal stem cells (MSCs) can be brought to the defect site to carry out the bone healing mechanism. The presence and properties of these scaffolds, which are primarily hydrogels such as Fibrin Glue (FG), can significantly influence cell survival and differentiation. The ability of 3D Nano printed β-TCP and MSCs with an admixture of fibrin glue to regenerate will have powerful implications in the reconstruction field of oral and maxillofacial surgery.

## Material and Methods

Twenty 3D printed β-TCP scaffolds, ten scaffolds ingrained with osteogenic stem cells with FG (group A), and ten scaffolds used as a control group with β-TCP scaffold and FG inoculation only (group B) were included in the study.

### 3D-Bioplotting of HA/TCP Scaffolds

**Figure 1:**
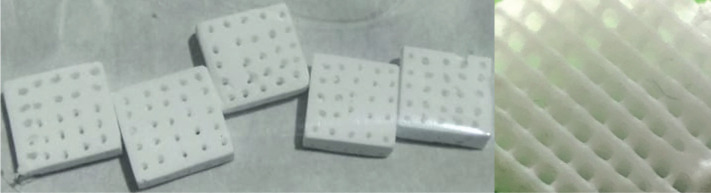
Three-dimensional printed β-TCP scaffold, 10×10×3 mm with 4-mm2 channels.

β-TCP (25μm particle size), and hydroxyapatite (HA) nanopowder (<200nm particle size) were utilized for 3D printing by bio plotting using a nozzle generating around 500 to 600 μm of pore diameter. The fabricated 3D scaffolds (Figure 1) were subjective of biocompatible certification with no induction of toxic or hazardous properties.

### Region of Bone marrow harvest

**Figure 2:**
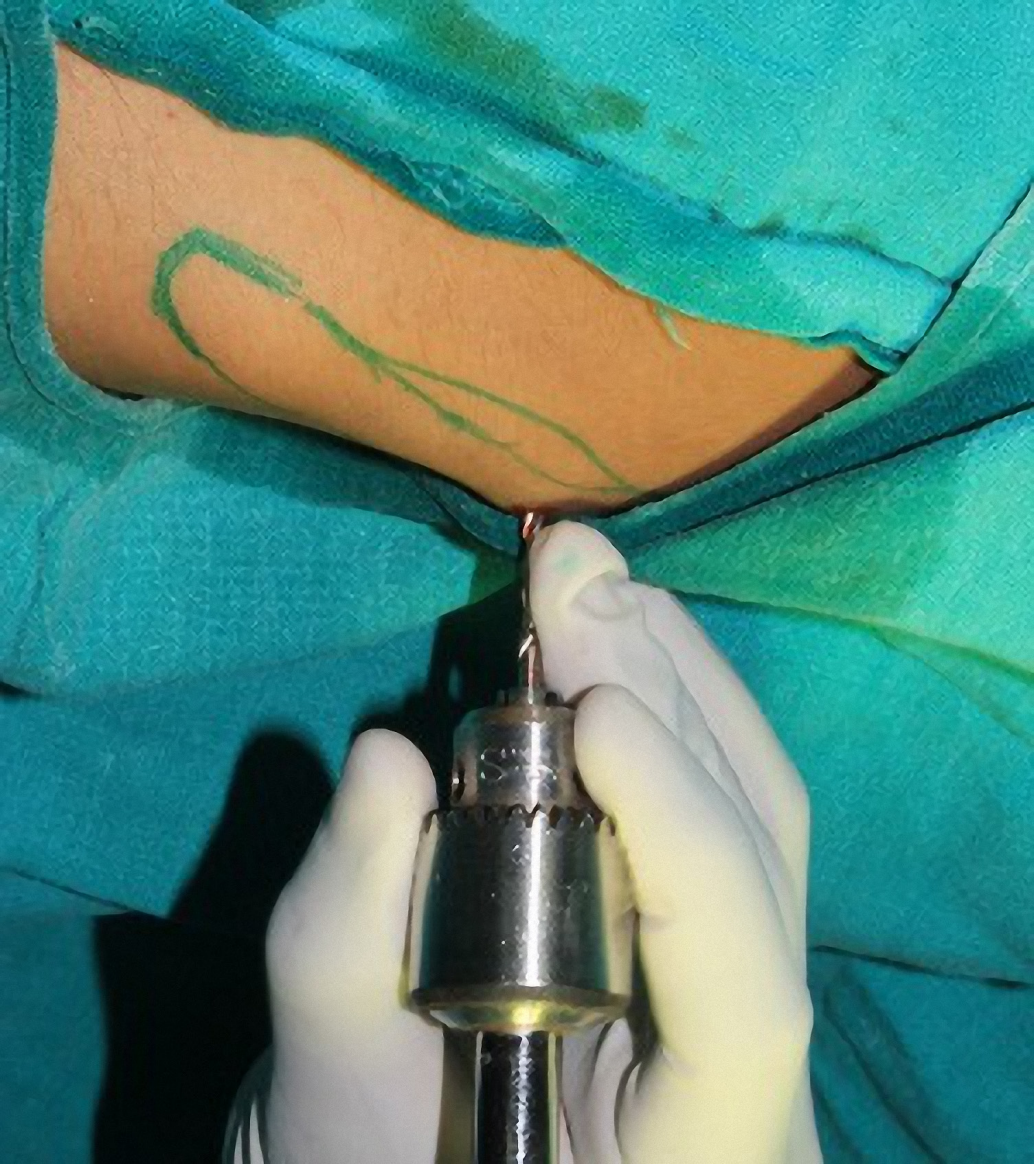
Bone marrow aspiration from the iliac crest.

**Figure 3 (A-F):**
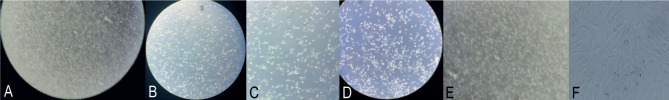
Viable osteogenic mesenchymal stem cells prepared for inoculation in different study scaffolds of Group A.

**Figure 4:**
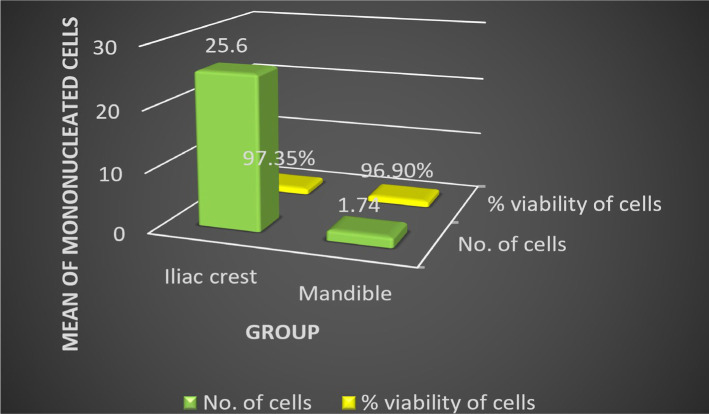
Mean comparison of concentration and viable cell percentage (%) from the iliac crest and mandible.

Bone marrow was harvested from the iliac bone and mandible, and further cell processing was completed. Seven samples of MSCs extracted from the iliac bone (Figure 2), and three samples of MSCs extracted from the mandible were obtained. Peripheral blood was also collected. These patients volunteered for the study, and informed consent was obtained before study onset. The intervening process was carried out as the need for the adjuvant surgical procedure followed by cell differentiation and viability count (Figure 3 A-F). A significant difference in the number of viable cells based on the site of extraction was evident (Table 1) (Figure 4).

**Table 1:** Outcome in Patients by the viability of autologous MSCs.

Case no.	Region of bone marrow harvest	The volume of bone marrow obtained in ml.	Density of cells
**1**	Iliac	15	2.9x108
**2**	Iliac	15	2x108
**3**	Iliac	15	1.8x108
**4**	Iliac	15	1.7x108
**5**	Iliac	15	3.4x108
**6**	Iliac	15	3.3x108
**7**	Iliac	15	2.9x108
**8**	Mandible	10	1.7 x 107
**9**	Mandible	10	1.8 x 107
**10**	Mandible	10	1.6 x 107

A dynamic growth medium infiltrated with an admixture of MSCs, FG with growth factors inoculated into the 3D printed scaffold in a sterile medium. Both static and dynamic culture conditions are utilized to study the cellular infiltration, migration, and proliferation of MSCs within the scaffolds. Radiographic and microscopic examinations were carried out to each of the scaffolds to evaluate and assess for opacities and formation of any bone-like tissues post culture after repeated changes in the nutrient medium at 2, 4, and 8 weeks. The post-inoculation and culture results were evaluated by histopathological assessment and micro radiographic scans in vitro.

## Results

Twenty 3D printed β-TCP scaffolds, ten scaffolds ingrained with osteogenic stem cells with FG (group A), and ten scaffolds used as a control group with β-TCP scaffold and FG inoculation only (group B) were included in the study.

### Macroscopic observations of the inoculated implants

The scaffolds of MSCs/ β-TCP fibrin glue admixtures formed by eight weeks were rigid and resisted compression. But only a shiny appearance and an elastic consistency at two weeks, which continued until eight weeks, appeared in FG/β-TCP scaffolds as they failed in the formation of such textures in comparison with MSCs/ β-TCP scaffolds (Table 2).

**Table 2:** Macroscopic surveys of the scaffolds showed that calcification occurred in the MSCs/ β-TCP fibrin glue admixtures at eight weeks but not in the control groups.

Group	Macroscopic	
	Positive (+)	Not Assessed (NA)
****Group A****	9	1
****Group B****	0	0

### Radiographic interpretation of study and control groups

**Figure 5:**
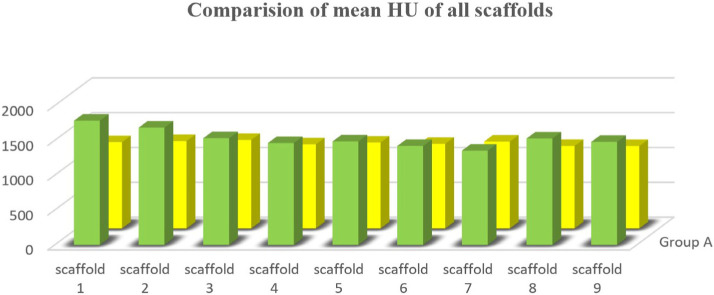
Comparison of mean HU of different scaffolds of the two study groups.

There was a significant increase in the basic Hounsfield units (HU) of Group A specimens with the control group B (Figure 5), which shows the positive response of inoculated MSCs with growth factors filled with fibrin glue and represents a favorable environment for bone regeneration and growth.

The results in terms of bone density by tomographic studies measured in HU of the test and control groups were analyzed using an independent sample t-test.

*Independent sample t-test*: group A showed a significant difference at week 8 in bone density in comparison with group B alone in different planes of study (all p values were less than 0.05), and the overall mean density scores also improved significantly in group A in comparison with group B.

### Histologic analysis and mineral detection

**Figure 6:**
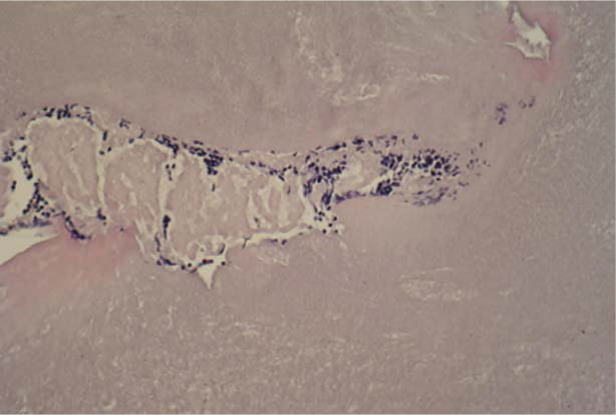
Initial fibrin, MSC/ β-TCP matrix.

**Figure 7:**
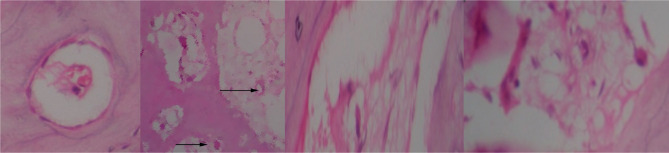
Histological examination of HE stains of Group A scaffolds.

Each scaffold was examined post culture after repeated changes in the nutrient medium at 2, 4 or 8 weeks and assessed for opacity and formation of any bone-like tissues manually. The samples were fixed and stained with hematoxylin and eosin (HE). Each specimen was blinded of identity and examined under a light microscope by the pathologist to determine the osseous tissue origin. In the initial period of inoculation, cell fragment aggregates were observed as whitish streaks in the apical part of the β-TCP, MScs fibrin matrix on histological sections (Figure 6). These are the cellular accumulations with fibrin matrix supported by β-TCP build-up (Figure 7).

**Figure 8:**
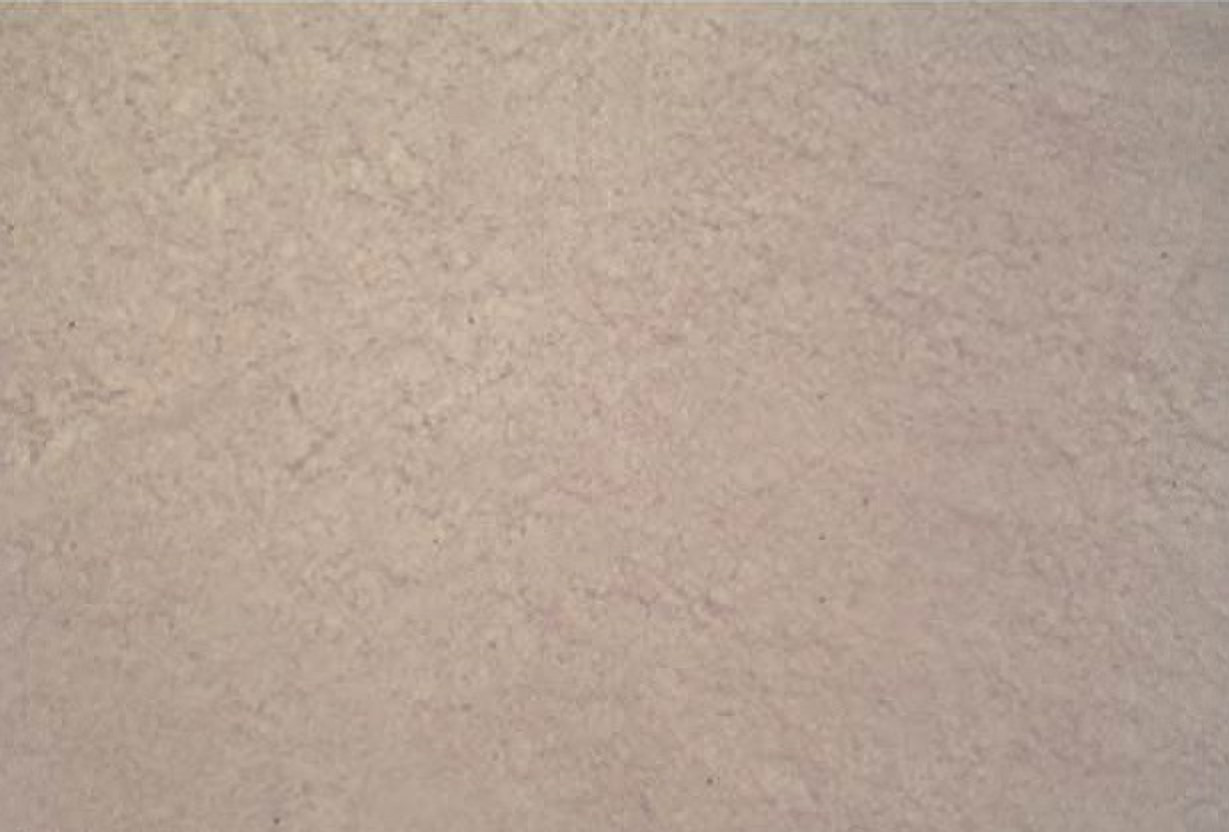
Group B scaffold post-study with empty pores and no cellular activity.

Group A scaffold with MSCs and FG, (10x magnification). The areas of interest, including an irregular matrix of osteoblastic rimming, osteoclast activity, are indicated by arrows. The osteoclast activity was positive and scattered along the edge of bone matrix vascular spaces, canals and haphazardly arranged osteocytes were found after eight weeks (Figure 7). In contrast, unloaded scaffolds from Group B showed no evidence of mesenchymal differentiation, and the pores were empty (Figure 8).

There was a significant difference associated with bone formation between Group A and Group B specimens with good bone regenerative results in the group inoculated by MSCs and FG.

## Discussion

The cortical and cancellous parts are the physiological constituents of the bone tissue. Regeneration and remodeling of osteogenic tissue are accomplished by different cells of connective tissue origin. Bone generation is mainly caused by osteoblasts, whereas osteoclasts cause bone resorption. The signaling and teamwork between the osteogenic cells maintain healthy bone through the remodeling process [1]. Medical implants and organ transplantation are the existing medical solutions for significantly injured or diseased bones [2]. However, complications like infection, irritation and morbidity associated with these procedures always challenge us to find new methods of transplantation and reconstruction. Tissue engineering is a promising and emerging field for the biological regeneration of desirable organ tissue using the principles of bioengineering and cell culture. This novel methodology uses regenerative cells from a person’s body and cultured in-vitro for seeding in a scaffold, which accentuates the division and differentiation of cells to form the three-dimensional tissues [3]. Gas foaming, particulate leaching, fiber bonding, phase separation, membrane lamination, melt molding, solvent casting, and emulsion freeze-drying were different traditional approaches to fabricate these scaffolds [4]. But this biological process of tissue biocompatibility is not provided by these scaffold fabrication methods. Additive manufacturing techniques are the major invention to fabricate such intricate structures successfully. These include 3D printing, stereolithography, fused deposition modeling, selective laser sintering, 3D Plotter [5]. Fabrication of complex and desirable inner structures is done with phase-change jet printing and low-temperature deposition manufacturing using computer-aided design (CAD) data [6]. The biocompatibility, osteoconductivity, solubility and scaffolding properties of remodeling of β-TCP make it a vital bone graft material in clinical use [7-9]. Most commonly, osteoconductivity is being integrated into β-TCP scaffolds is by incorporation of bone-derived growth factors like BMP-2, IGF-1 and 2, VEGF [10, 11]. However, challenges of controlled release and long-term viability of these growth actors are of significant concern in scaffold incorporation [10].

Cell proliferation within the scaffold depends on many properties of the scaffold, which includes the porous nature of the scaffold, surface area/volume ratio, thickness, anisotropy, cross-sectional area, permeability, and interconnection of the compartments. The release of oxygen and nutrients requires 100 to 350 μm of macropores and 5 to 10 μm of micropores for cell adhesions within a perfect scaffold [12, 13]. Many studies proposed on optimizing the porosity value of these scaffolds with a compressive modulus of the host tissue [14]. However, some studies proposed there is a need for an equilibrium of mechanical properties between the recipient host tissue and scaffold to remodel into a new tissue [15].

Recent advances have taken place in the field of tissue engineering and scaffold architecture. Since traditional scaffold fabrication by traditional methods was not efficient for tissue engineering abilities, 3D printing has led researchers to fabricate scaffold with the required physical properties. Chua et al. developed a library with diverse polyhedral shapes to form the unit element of the scaffold structure and has chosen the unit cells to form a scaffold structure based on their complexity, mechanical integrity, and gap-filling properties [16]. MSCs have a vital role to play in tissue engineering to regenerate desirable tissue defects. The availability of MSCs in bone marrow aspirates are at a concentration of 10-100 MSC per 1x106 BM cells [17-21], and high cell numbers are needed to accelerate bone remodeling and regeneration depending on the size of the defect of the bone tissue, which necessitates efficient isolation of MSCs [17, 22]. Clinical investigations are in a trial for cardiac and orthopedic purposes requiring instant stem cell therapy [19, 23-26]. the current use of appropriate injectable MSCs is reliable on a sufficient number and concentration of MSCs separation without expansion steps [19].

Additional laboratory analysis is required to identify these MSCs within the cellular fraction; therefore, the complete cellular fraction can be used for cell therapy. The injection of a mixed cell population of nuclear cells influencing the performance of tissue regeneration lacks evidence [19, 23]. Literature might also postulate that white blood cells can cause an inflammatory reaction with negative side effects [27]. And also, cell signaling and interaction with other precursor cells within the cellular fraction might be beneficial to the tissue of regeneration [28]. The growth of colony-forming unit fibroblasts (CFU-F) in culture is supported by the mixture of mononuclear cells, lymphocytes, and polynuclear leukocytes [29]. Therefore, this explains the increased MSC count from the VOL RED group, as described by Kasten et al. [30]. A promising alternative in regenerative medicine has emerged by the use of mesenchymal progenitor cells in cell-based strategies of bioengineering. In the past, the bone repair was suggested using bone marrow mesenchymal cells, osteoblasts from the axial skeleton, and osteoblasts from orofacial bones. Cell activity on HA- β-TCP scaffolds was evaluated using human bone stem cell lines and observed that the optical density value and percentage viability of cells increased with culture time for scaffolds with 10 and 20% HA concentrations. In contrast, a decrease in percentage viability was observed for scaffold with a 30% HA concentration. Results suggest that the b-TCP scaffold sintered at 1400°C with a 20% HA concentration is more beneficial to bone cell growth.

The present study demonstrates a significant acceleration in angiogenesis for bone regeneration by the channeled porous scaffold, facilitating nutrient diffusion without losing mechanical strength and promoting cell infiltration and proliferation. However, primarily limitations of in-vitro osteogenic promoting properties have to be studied yet. Secondly, the deep investigation of the signaling pathway regulating cellular migration and angiogenesis has to be done and investigated in the forthcoming studies. Third, in vivo vascularization and bone regeneration by multiple channeled porous β-TCP scaffolds with MSCs and growth factors needs to be verified in the next step. Therefore, future works should focus on 3D printed scaffolds and MSCs in promoting angiogenesis and osteogenesis in vivo. The physiologic fibrin matrix serving as a platform provided by FG and accelerates angiogenesis in the fibrin membrane. Larger osseous defects have of particular interest in this aspect. Perhaps such healing requires stem cells and their ability for conversion to the osteoblastic precursor. MSCs from bone marrow contribute to the regeneration of whole type bone cells and many other tissues.

Fibrin matrix is optimal support to transplanted MSCs for obtaining osseous defect regeneration. In our study, twenty 3D printed β-TCP scaffolds, ten scaffolds ingrained with osteogenic stem cells with FG (group A) and ten scaffolds used as a control group with β-TCP scaffold and FG inoculation only (group B) were included. The scaffold of MSCs/β-TCP fibrin glue admixtures at eight weeks were hard and resisted compression, but FG/β-TCP with no such hard textures and appeared shiny and elastic at two weeks until eight weeks. Radiographic interpretation of this scaffolds showed that calcification occurred in the MSCs/β-TCP/FG admixtures eight weeks but not in the control groups. There was a significant association with the macroscopic appearance of bone on the usage of β-TCP scaffold/MSC’s and fibrin glue than using fibrin glue alone (P=0.035). The examination and evaluation involved both morphological and bone density analysis in HU. The CT density scale with HU was useful in identifying and differentiating structures and tissues. The findings have statistical significance. Group A showed a significant difference at week 8 (as suggested by mean bone density values) in bone density in comparison to group B in different planes of study (all p-values were less than 0.05). The overall mean density scores also significantly improved in the test group A in comparison with the control group B. Tanaka et al. established in 2014 an evaluation system for bone formation and monitoring β-TCP resorption in opening high tibial osteotomies, showing complete resorption of β-TCP with 75% porosity and replacement by bone [31]. Our study also showed significant bone formation in cases with Stem cells and FG, at 8 weeks. Histological studies and analysis showed that each scaffold was examined post culture after repeated changes in the nutrient medium at 2, 4 or 8 weeks and assessed for osteoid tissue formation and opacities. The samples were fixed and stained with hematoxylin and eosin. These specimens were examined by a pathologist under a light microscope, blinded to the identity of each specimen, and asked to determine the presence or absence of bone formation. Group A scaffolds integrated with MSCs and fibrin glue (10x magnification) showed osteoblastic riming, larger vascular spaces, canals, and haphazardly arranged osteocytes found after four weeks (A). Group B scaffolds showed no evidence of MSCs differentiation, and the pores were all empty (B). Yoichi Yamada et al. stated that MSCs/β-TCP/FG admixtures resulted in successful bone regeneration and reconstruction of bony defects by minimally invasive means of generating autogenous bone [32].

Our study results were in concurrence with this study. Exploration of the growth factors contained within platelets and the ability of biological enhancement of continuity bone grafts achieved. Platelet-rich fibrin (PRF) is a platelet concentrate developed in France by Choukroun et al. since 2000 [33]. The use of PRF in oral and maxillofacial surgery is a current and interesting trend. These basic research studies consisting of a large number of production protocols were developed, marketed, and tested with custom made protocols. PRF can be used as the sole biomaterial [32, 34] or combined with different bone substitutes [35, 36].

The use of bone graft materials along with PRF accelerates bone regeneration. PRF is stable and strong after application and does not dissolve quickly and slowly remodels as a natural blood clot. It is postulated that PRF consisting of growth factors could accelerate the process of bone regeneration by attracting cell migration, differentiation, and genesis of microvessels through the fibrin scaffold of PRF. In our study, the combination of MSCs with only FG and β-TCP resulted in bone formation. From the results of this study, FG provides an appropriate environment for the proliferation and differentiation of cells in vitro. The MSCs were able to undergo many passages without losing their osteogenicity [17]. Bone formation and repair are achieved in clinical settings by generating a large number of potential osteogenic cells using the expansion of MSCs [31]. A promising alternative to autologous bone grafts in maxillofacial skeletal repair will be the tissue engineering process of the inoculation of autogenous properties in the β-TCP scaffold by MSCs and growth factors. Therefore MSCs with fibrin scaffolds will have promising applications in the future of regenerative medicine. However, in vivo studies and different challenges of MSC interactions with growth factors and fibrin matrix are still to be addressed regarding tissue regeneration in regards to advancements in the clinical utility and safety of clinical tissue regeneration therapy.

## Conclusion

Maxillofacial skeletal repair via tissue engineering by β-TCP scaffold and MSCs remains the most promising and effective method for reconstruction of skeletal defects, pausing the morbidity caused in autologous bone graft harvest and take-up. Bone marrow MSCs and MSCs with fibrin scaffolds will likely have extensive applications in the future. However, several challenges remain before the use of this therapy in everyday clinical practice. A greater understanding of the mechanisms of interaction among MSCs, growth factors, and fibrin matrix regarding tissue regeneration is needed to advance the clinical utility of this therapy. Given that the potential risks of applying stem cells, additional animal studies are required on the safety of clinical tissue regeneration.

## Conflict of Interest

The authors declare that there is no conflict of interest.
